# Data for comparison of chlorine dioxide and chlorine disinfection power in a real dairy wastewater effluent

**DOI:** 10.1016/j.dib.2018.03.117

**Published:** 2018-03-29

**Authors:** Maliheh Akhlaghi, Amin Dorost, Kamaladdin Karimyan, Mohammad Reza Narooie, Hooshmand Sharafi

**Affiliations:** a Students Research Committee, Gonabad University of Medical Sciences, Gonabad, Iran; b Students Research Committee, Torbat Heydariyeh University of Medical Sciences, Torbat Heydariyeh, Iran; c Environmental Health Research Center, Kurdistan University of Medical Sciences, Sanandaj, Iran; d Department of Environmental Health Engineering, School of Public Health, Tehran University of Medical Sciences, Tehran, Iran; e Department of Environmental Health, School of Public Health, Iranshahr University of Medical Sciences, Iranshahr, Iran; f Students Research Committee, Kermanshah University of Medical Sciences, Kermanshah, Iran

**Keywords:** Disinfection, Chlorine dioxide, Chlorine, Total coliform, Fecal coliform

## Abstract

Disinfection of water refers to a special operation that is doing to kill or disable causative organisms (i.e. Pathogens) and in particular, intestinal bacteria. The aim of this pilot study is comparison of disinfection power of Chlorine dioxide and chlorine in a real dairy wastewater effluent. In this regard, firstly prepared two 220-l tanks made of polyethylene as reaction tanks and filled by effluent of a dairy wastewater treatment plant. Both tanks were equipped with mechanical stirrer. Then a Diaphragm dosing pumps with the maximum capacity of 3.9 l per hour were used for the chlorine dioxide and chlorine (Calcium hypochlorite) 0.5 up to 3 ppm injection. Residual level of Chlorine dioxide and Chlorine were measured by portable photometric method DT4B kit, Germany. Finally, the Multiple-Tube Fermentation, Brilliant Green Bile Broth (BGB) and Eosin methylene blue Agar (EMB) technique was used for microbial analysis and the results were reported as the most probable number index (MPN) respectively. The data showed that the residual of chlorine dioxide could stood more active than residual of chlorine in the aqueous environment significantly. Therefore, Use of chlorine dioxide is more effective than chlorine for removal fecal and total coliform from dairy wastewater effluent.

## Specifications Table

**Table:** 

Subject area	Environmental Sciences
More specific subject area	Chemistry
Type of data	Tables
How data was acquired	The residual level of Chlorine dioxide and Chlorine were measured by portable photometric method DT4B kit, Germany. Finally, the Multiple-Tube Fermentation, Brilliant Green Bile Broth (BGB) and Eosin methylene blue Agar (EMB) techniques were used for microbial analysis and the results were reported as the most probable number index (MPN) respectively.
Data format	Raw, analyzed
Experimental factors	The dairy wastewater effluent disinfected by chlorine dioxide and chlorine and then the microbial activity of it was evaluated by MPN method.
Experimental features	The residual level analysis of Chlorine dioxide and Chlorine and also microbial activity were measured according to the standards for water and wastewater treatment handbook.
Data source location	Gonabad, Mashhad province, Iran
Data accessibility	Data are included in this article

## Value of the data

• Despite the advantages of chlorine dioxide in water treatment, no study has compared the disinfection power of chlorine dioxide and chlorine so far. To address this gap, the result and data of this study is the first attempt comparing the disinfection power of dioxide and calcium hypochlorite in removing fecal coliform and total coliform in the effluent from dairy wastewater treatment plant. It also examines the extent to which chlorine dioxide and calcium hypochlorite can remain active.
• Due to limited studies in the study area, the data of this study can help to better understand the disinfection power of the Chlorine dioxide and Chlorine over effluent of a dairy wastewater treatment plant and provide further studies.
• The obtained data showed that chlorine dioxide using a recommended dose (1.5 ppm) in contact time 30 min is more effective than chlorine (calcium hypochlorite) on the removal of fecal coliform and total coliform from dairy wastewater effluent.
• The data from this study can provide a basis for future studies in relation to disinfection practices and the selection of a suitable disinfectant for industrial wastewater treatment.

## 1 Data

In this study, we conducted all tests and procedures explained on the seven samples collected from two water tanks. To one of the tank, we injected chlorine dioxide while to another we injected calcium hypochlorite. Table 1 shows the count for Coliforms and fecal streptococcus bacteria, heterotrophic in tanks containing different amount of chlorine dioxide and Table 2 summarized the count for Coliforms and fecal streptococcus bacteria, heterotrophic in different amount of chlorine. The results presented in Table 1 are shown that in all cases the bacteria count was less than 1 MPN. These finding show that after the injection of chlorine dioxide, water has been cleaned. The results presented in Table 2 indicate contamination of the samples, despite the injection of chlorine in different amounts.

**Table 1:** Microbial analysis after chlorine dioxide injection, 30 min.

**No**	**ClO**_**2**_**, ppm**	**Heterotrophic bacteria CFU/ml**	**Total coliform MPN/100 ml**	**Thermal coliform MPN/100 ml**	**Streptococcus**	**Result**
1	0.50	26	< 1.1	0	< 1.1	Clean
2	0.75	14	< 1.1	0	0	Clean
3	1.00	8	< 1.1	0	0	Clean
4	1.50	0	0	0	0	Clean
5	2.00	0	0	0	0	Clean
6	2.50	0	0	0	0	Clean
7	3.00	0	0	0	0	Clean

**Table 2:** Microbial analysis after calcium hypochlorite injection, 30 min.

**NO**	**Ca(ClO)**_**2**_**, ppm**	**Heterotrophic bacteria CFU/ml**	**Total coliform MPN/100 ml**	**Thermal coliform MPN/100 ml**	**Streptococcus**	**Result**
1	0.50	120	20	5	< 1.1	polluted
2	0.75	86	13	2	< 1.1	polluted
3	1.00	41	< 1.1	2	< 1.1	polluted
4	1.50	27	< 1.1	1	< 1.1	polluted
5	2.00	7	< 1.1	1	< 1.1	polluted
6	2.50	1	< 1.1	0	< 1.1	Clean
7	3.00	0	0	0	< 1.1	Clean

The residual level of dioxide (Table 3) and free chlorine (Table 4) after 30 min, was recorded at 24 and 48 h intervals. Comparing the results in Tables 3 and 4 show the residual of chlorine dioxide can stay significantly more active than chlorine residual in the environment.

**Table 3:** Active chlorine dioxide remaining at different times, ppm.

**No**	**Initial ClO**_**2**_	**30 min**		**24 h**	**48 h**
		**ClO**_**2**_	^**−**^**ClO**_**2**_	**ClO**_**2**_	**ClO**_**2**_
1	0.50	0.00	0.00	0.00	0.00
2	0.75	0.32	0.16	0.00	0.00
3	1.00	0.40	0.25	0.00	0.00
4	1.50	0.70	0.25	0.00	0.00
5	2.00	1.20	0.19	0.00	0.00
6	2.50	1.50	0.25	0.32	0.15
7	3.00	1.75	0.40	0.75	0.20

**Table 4:** Active chlorine remaining at different times, ppm.

**No**	**Initial Ca(ClO)**_**2**_	**30 min**	**24 h**	**48 h**
1	0.50	0.00	0	0
2	0.75	0.00	0	0
3	1.00	0.25	0	0
4	1.50	0.67	0	0
5	2.00	1.10	0	0
6	2.50	1.60	0	0
7	3.00	1.80	0	0

## 2 Experimental design, materials and methods

### 2.1 Pilot equipment

In these pilot two 0.22 m^3^ tanks made of polyethylene was used as reaction tanks. For the purpose of uniformity, both tanks were equipped with mechanical stirrer. Diaphragm dosing pumps (ProMinent® CONCEPT plus) with the maximum capacity of 3.9 l per hour was used for the injection. To produce chlorine dioxide, preparation and administration package of chlorine dioxide (ProMinent LegioZon, Germany) with a capacity of 5 g per hour was used. Residual level of dioxide and chlorine were measured by DT4B (DULCOTEST®, Germany) through photometric method (Photometric accuracy was 3% full Scale). The experiments were performed at 25 ± 2 °C [1].

### 2.2 Preparation and microbial analysis of samples

A calcium hypochlorite powder 68% was used to prepare chlorine in solution. To prepare chlorine dioxide from sodium chloride 25% (7.5%) and chloric acid 37% (9%) Merck, Germany was used. The multiple-tube fermentation technique (here 10 tubes) was used for microbial analysis, the results were reported as the most probable number index (MPN) respectively [1], [2], [3]. In this technique, specific volumes of sample are placed on appropriate culture medium into the test tubes. This method has three stages: presumptive stage, confirmed stage, and completed test. In the presumptive stage, the medium used was lactose broth. On culture medium in fermentation tube, Durham was used to show fermentation gas after adding water; the samples were incubated for 48 h at 35 ± 0.5 °C. In the confirmed stage, we used only presumed positive tubes (tubes showing gas formation in the Durham tube). We transferred medium from those tubes to the BGB by a platinum loop. The samples were then incubated for 48 h at 35 ± 0.5 °C. We also used the EC culture broth to detect E. coli bacteria. The injection method was similar to BGB's. The tubes were incubated for 24 h at 35 ± 0.5 °C. In the completion test stage, we streaked samples from confirmed positive test to EMB Agar plates by platinum and plates were incubated upside down at 35 ± 0.5 °C. We also used Azide Dextrose broth to detect *faecal streptococci* and the results were reported as MPN table. Heterotrophic bacteria was detected by plate count using pour plate and presented by colony forming unit (Colony Forming Unit) respectively [1], [2], [3]. The residual level of Chlorine dioxide and Chlorine and also microbial analysis were measured according to the standards for water and wastewater treatment handbook [4], [5], [6], [7], [8].

### 2.3 Procedure

The samples was prepared from dairy industry effluent that was treated by activated sludge process (extended aeration), Table 5. At first, we conducted microbial analysis on raw wastewater. We added the same amount from each disinfectant to the tanks then we equally added microbial contamination to both tanks. We used mechanical stirrer 900 rpm for 30 s to uniform the solutions in each tank and then we recorded the residual level of chlorine and chlorine dioxide after 30 min. Samples for microbial analysis were collected from different concentrations of dioxide and calcium hypochlorite after 30 min and were analysed using the multiple-tube fermentation technique. The analysis included three stages: the presumptive, confirmed and completed. We measured the residual level of chlorine and chlorine dioxide and chlorine at different three time intervals: after 30 min, 24 and 48 h. During the experiments, the room temperature was 28 ± 3 °C and the water temperature was 25 ± 2 °C and the pH levels were between 6.9 and 7.3.

**Table 5:** Characterization of the real dairy effluent.

Turbidity (NTU)	DO (mg L^−1^)	Temperature (°C)	Microbial (CFU mL^−1^)
2–5	6.5–7.8	20–25	2300–9300
