# Supplementary material for: Data for comparison of chlorine dioxide and chlorine disinfection power in a real dairy wastewater effluent
Source: Data Brief. 2018 Mar 29;18:886–90. doi: 10.1016/j.dib.2018.03.117 (PMC5996494; doi:10.1016/j.dib.2018.03.117)
Supplement: Supplementary file 1 — Supplementary material [file mmc1.docx]

Dear editor(s)

Data in Brief

Greetings!

At this time, this article with entitled ***” Data on Comparison of Disinfection power of the Chlorine dioxide and Chlorine over Effluent of a Dairy Wastewater treatment plant*** “ has not been published or submitted in any other journals. we hope that the results of this research will be of interest to you. None of the authors of this paper have any conflicts of interest to declare.

Sincerely yours

Corresponding author

Hooshmand Sharafi
